# The chromatographic constitution of andiroba oil and his healing effects, compared to the LLLT outcomes, in oral mucositis induced in golden Syrian hamsters: a new treatment option

**DOI:** 10.18632/oncotarget.28338

**Published:** 2023-01-12

**Authors:** Jessica T. Gomes, Ana Márcia V. Wanzeler, Sergio M.A. Júnior, Rosa Helena F. Chaves Soares, Carolina P. de Oliveira, Emanuelle de M. Rodrigues, Bruno M. Soares, Diego D.F.A. Alcantara, Rommel M.R. Burbano, Fabrício M. Tuji

**Affiliations:** ^1^Department of Dentistry, Federal University of Pará, Guamá, Belém, Pará 66075-970, Brazil; ^2^Department of Medicine, University Center of Pará, Souza, Belém, Pará 66613-903, Brazil; ^3^Department of Genetics, Laboratory of Human Cytogenetics, Federal University of Pará, Guamá, Belém, Pará 66075-970, Brazil

**Keywords:** phytotherapeutic drugs, medical oncology, stomatitis, wound healing, low-level light therapy

## Abstract

The oral mucositis is a mucosal alteration that usually arises from oncological treatments, such as chemotherapy, and it is characterized as an inflammatory process. The aim of this study is to demonstrate the chromatographic constitution of Andiroba oil, comparing and evaluating Andiroba oil and laser scarring efficiency in treatments of oral mucositis in hamsters. These animals were submitted to 5-Fluorouracil. A total of 122 animals were used, randomized and divided into the following groups: (a) positive control; (b) laser associated to andiroba oil; (c) laser; (d) andiroba oil; (e) negative control; (f) cyclophosphamide (genotoxicity control). The induction of oral mucositis occurred by the administration of intraperitoneal Fluorouracila (60 mg/kg) and trauma to the mucosa. The laser protocol was performed once a day and the andiroba oil applied 3 times a day (1,5 ml/day). The mucosae were photographed and removed for clinical and histopathological analysis on day 4, 8, 12 and 15. The analysis was based in OM severity, in specific scoring for the clinical and histopathological aspect. Toxicity was evaluated on day 15 using comet assay and it was performed by variant DNA damage parameters. The data were analysed using analysis of variance (ANOVA) Tukey post*-test* and Kruskal–Wallis Dunn post*-test*. The “andiroba oil” and “laser” groups presented better results when compared to the control groups and the treatment associations. The andiroba oil presented the best scarring results, even considering its efficiency proximity to the laser treatment. Andiroba and laser, separately, did not present genotoxicity, however their association evidences damage to DNA.

## INTRODUCTION

The oral mucositis (OM) is a mucosal alteration that usually arises from oncological treatments, such as chemotherapy, and it is characterized by an inflammatory process that promotes the development of painful ulcerations in the oral mucous membranes [1].

Among the most used chemotherapeutic drugs in antineoplasic therapies, the 5-Flourouracil (5-FU) can result in development of OM as a side-effect of its application [2]. Thus, the execution of a preventive and curative treatment to reduce OM incidence seems to be necessary. The regular medical interventions are palliative conducts related to releasing the OM symptomatology [3], such as the use of cryotherapy [4], topical anesthetics, antifungals, antiseptics, antivirals, antibiotics, anti-inflammatory drugs inhibitors of COX-2 and/or prostaglandin E2, protective agents of the mucosa, vitamins E and A [5], use of low-level laser therapy [6–9], and phytotherapy [10–12].

The use of phytotherapy shows satisfactory results in OM treatments, such as: chamomile [13–15], bilberry extract [16], Hydroalcoholic extract of Carumcarvi L [17], propolis [18] and eucalyptus hydroalcoholic extract [19]. Even though that herbal medicine is a very ancient practice, the studies related to the medical use of plants and its acceptance became more popular in the last years [20]. The plants are processed and their active substances are extracted and transformed into essential oils, which can be administered topically [21] or systemically [22]. The topical formulation presents higher acceptability by the patients, the prompt absorption and rapid onset of action [23]. However, the practice of phytotherapy is still hindered due to the lack of technical-scientific documentation and studies demonstrating clear clinical evidences and advantages of its use [20], as well as its pharmacological effects, genotoxicity and quality control, which must be overcome [24–29].

Andiroba (*Carapa guianensis Aubl*) is a plant that belongs to the *Meliaceae* family [30], and it will be found in Amazon region [31–34], southern Central America, Colombia, Venezuela, Suriname, French Guiana, Brazil, Peru, Paraguay and the Caribbean Islands [35, 36]. In Brazil, it’s found in North (states of Acre, Amazonas, Amapá, Pará) region [37] and Northeast (Maranhão state) regions [38]. The tree evidences good pharmacological properties considering its variety of components, such as flowers, leaves and stems extracts, but the oil demonstrated better medicinal effects when obtained by seeds pressing [39]. The seed oil presents yellowish and thick consistency and a very bitter taste (Meliacin presence in the composition), and are located into 6–8 cm fruits [40].

Andiroba’s oil is composed by saponifiable (95%) and unsaponifiable substances (2 a 5%), and within the saponifiable profile, it is important to highlight the presence of essential fatty acids, such as oleic, palmitic, stearic and linoleic acids, which demonstrate higher notoriety in medical applications [36, 37]. Studies have shown that essential fatty acids are the catalyzing agents of the healing process [38], stimulating cell proliferation, collagen production, antimicrobial, anti-inflammatory and antioxidant action [41–49]. The andiroba’s oil shows many therapeutic finalities, due its natural repellent [49], dermatological properties [50, 51], as well as its excellent anti-inflammatory, antibacterial performance, wound healing activity [52, 53], antiparasitic, insecticide [54, 55], anti-allergic effect [56], and antinociceptive responses [57, 58].

The andiroba extract in wounds provides acceleration in healing process, with increase of contraction rate and local re-epithelialization, resulting in the complete closure of wounds [52]. Cicatricial effect was evaluated in different concentrations of andiroba oil (*Carapa guianensis Aubl*) in wound healing (oral mucositis), demonstrating an excellent cicatricial effect and acceleration of this process [28]. A clinical research in cancer patients evaluating the potential of andiroba oil cicatricial compared to low-power laser showed a significant improvement in the clinical picture of oral mucositis lesions and the symptomatology [29].

The use of laser therapy is an alternative resource for treating OM due to its biostimulation potential which increases the ulcerated area healing process, promoting physiologic responses, modulating the inflammation, accelerating wound healing and relieving pain. As a non-invasive treatment, it’s a therapy method with large acceptance by most patients, also, because it works in lessen the pain symptomatology since the first use. Also, it potentializes the complete tissue local revascularization until the fifteenth day and an intense tissue healing already in the second to fourth day. The Multinational Association of Supportive Care in Cancer/International Society of Oral Oncology (MASCC/ISOO) published a clinical practice guidelines for mucositis recommended the use of laser therapy in patients undergoing anti-cancer therapy [59, 60].

The low-level laser therapy (LLLT) is the best standard treatment and the most efficient method in treating OM. Similarly, the andiroba oil presents great potential for the treatment of inflammatory diseases. Thus, this study aims to evaluate the healing and toxicological effects of andiroba oil, compared to the LLLT outcomes, observing if andiroba presents a similar/higher potential than the LLLT.

## RESULTS

### GC-MS analysis of the oils

To verify the andiroba oil characterization, a layout of its chromatographic profile was made using gas chromatography, in which 12 saponifiable compounds were found, by the lipidic analysis. Among the compounds identified in the andiroba oil, the most expressive percentages were oleic acid (47.33%), palmitic acid (31.46%), linoleic acid (8.98%) and stearic acid (7.12%), as described in Table 1.

**Table 1:** Composition of the fatty acids present in andiroba oil (C. *guianensis*)

**Real Time**	**Compounds**	**Composition %**
7,199	Heptanoic acid	0,3989
13,674	Lauric acid	1,1572
15,883	Myristic acid	0,6394
17,982	Palmitic acid	31,4641
18,193	Palmitoleic acid	0,9184
18,862	Heptadecanoic acid	0,1154
19,845	Stearic acid	7,1279
20,064	Oleic acid	47,3356
20,428	Linoleic acid	8,9869
20,966	Linolenic Acid	0,2501
21,530	Arachidic acid	1,0897
23,562	Behenic acid	0,5164

**Figure 1:**
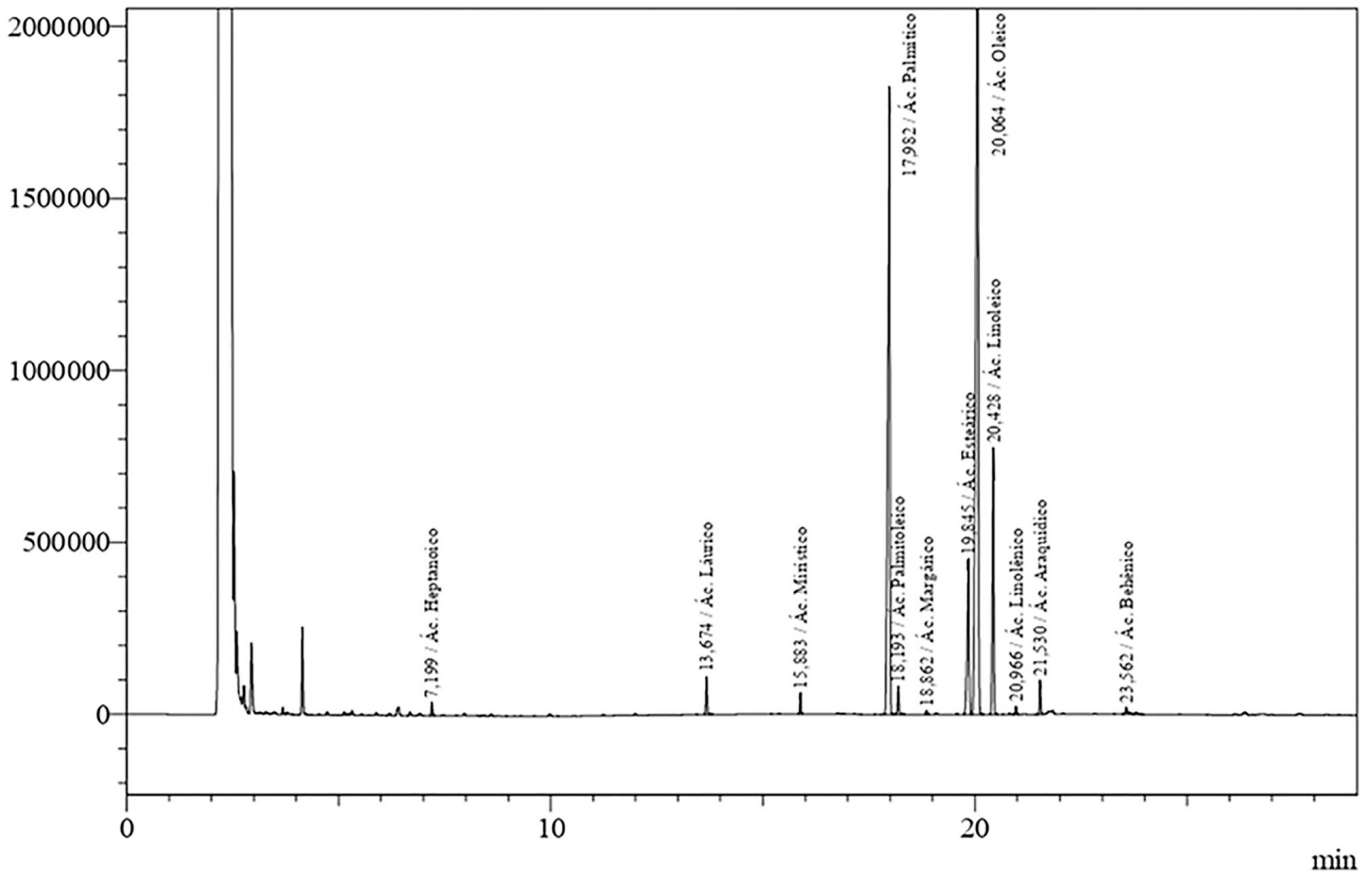
Chromatographic profile of *C. Guianensis* in natura oil. Presentation of the lipid composition based on Table 1.

To identify the most important lipidic compounds inside the andiroba oil, it was submitted to a chromatographic profile (Figure 1).

### Histopathological and clinical analysis

**Figure 2:**
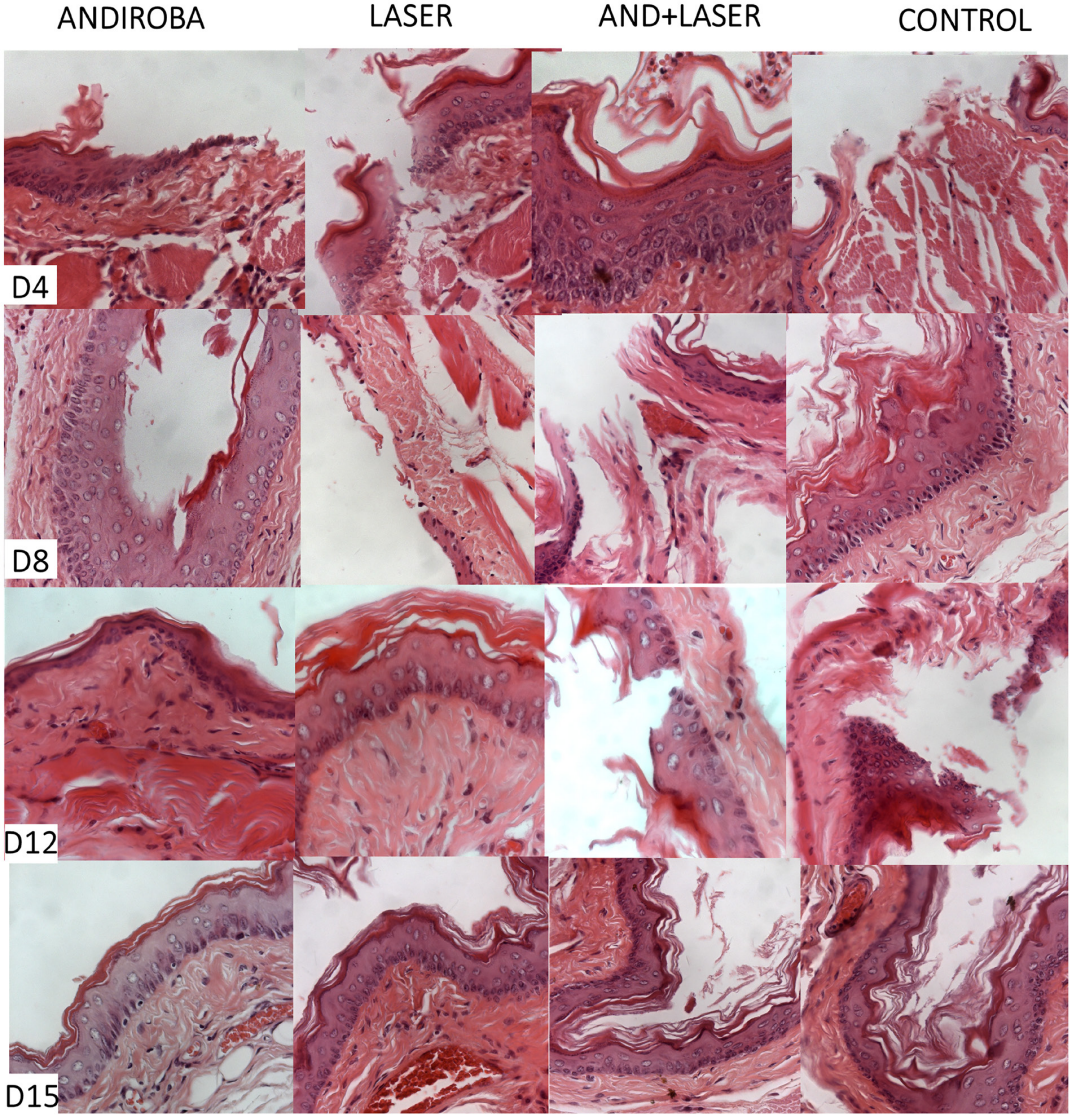
Histopathological evaluation of oral mucositis on days 4, 8, 12 and 15.

**Figure 3:**
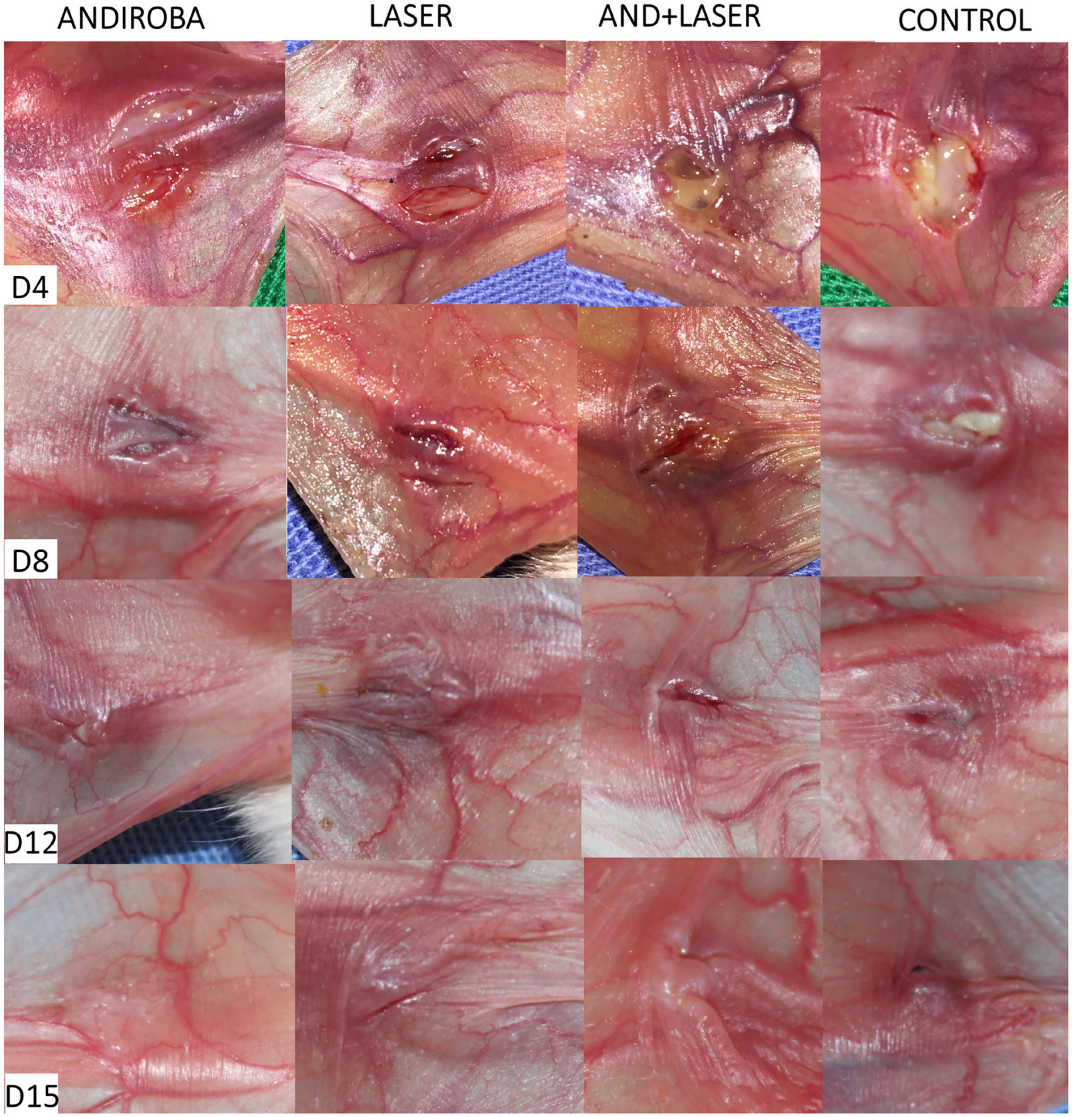
Clinical evaluation of oral mucositis in hamsters on days 4, 8, 12 and 15.

The clinical and histopathological evaluation of mucositis was performed by two previously trained examiners for each analysis. The scores used in research were based in “Lima et al. (2005) [61] modified” classification. To clarify clinical and histopathological results, we provide the Figures 2 and 3, corresponding to days 4, 8, 12 and 15 and their respective treatments.

The images and glass slides analyzed corresponded to the mucositis and their respective treatments on days 4, 8, 12 and 15, as expressed in Table 2.

**Table 2:** Description of the analyzes performed for clinical evaluations of oral mucositis in hamsters treated on days 4, 8, 12 and 15

	**Group**	***N***	**Mean**	**Median**	**SD**	**Variance**	**Minimum**	**Maximum**
D4	ADB^*^	14	2.29	2.00	0.469	0.220	2	3
	LASER^*^	14	2.29	2.00	0.726	0.527	1	4
	ADB + LASER	14	2.57	3.00	0.514	0.264	2	3
	CONTROL	14	3.07	3.00	0.616	0.379	2	4
D8	ADB^*^	14	1.71	2.00	0.611	0.374	1	3
	LASER^*^	14	1.86	2.00	0.864	0.747	1	4
	ADB + LASER^*^	14	1.93	2.00	0.616	0.379	1	3
	CONTROL	14	2.64	3.00	0.633	0.401	2	4
D12	ADB	14	1.50	1.00	0.650	0.423	1	3
	LASER	14	1.50	1.50	0.519	0.269	1	2
	ADB + LASER	14	1.64	1.50	0.745	0.555	1	3
	CONTROL	14	1.71	2.00	0.726	0.527	1	3
D15	ADB	14	1.14	1.00	0.363	0.132	1	2
	LASER	14	1.14	1.00	0.363	0.132	1	2
	ADB + LASER	14	1.21	1.00	0.426	0.181	1	2
	CONTROL	14	1.21	1.00	0.426	0.181	1	2

Kruskal Wallis; ^*^ *p* < 0.05; Control (Positive control group); Andiroba (andiroba group); Laser (laser group); Adb + laser (andiroba associated laser group); D4 (4 experimental day); D8 (8 experimental day); D12 (12 experimental day); D15 (15 experimental day).

In the clinical analysis on days 4 and 8, the “laser” group and “andiroba” group presented significant statistical differences when compared to the control group (*p* < 0.05, respectively), evidencing a possible similarity in their clinical efficiencies. For the better enlightenment of this difference, we provide the Figure 2.

In the clinical analysis, significant difference was noticed (*p* < 0.05) between “laser” and “andiroba” groups compared to positive control group. By analyzing the means of positive control group (3.07) and “laser associated to andiroba” group (2.57), the groups “laser” and “andiroba” presented lower means (2.28), evidencing similar clinical results on day 4 for both treatments. On day 8, the positive control group (2.64) and “laser associated to andiroba” group (1.92) demonstrated higher means in comparison to “laser” (1.85) and “andiroba” (1.71) groups. On days 12 and 15, there was no significative difference to be found when comparing the groups, however the scores presented higher means in positive control group, followed by the treatments association – laser and andiroba.

On day 4 of histopathological analysis, it was observed that groups “andiroba” and “laser” presented significative statistical differences in comparison to control group (*p* < 0.05), which had the higher score means (4.07) in comparison to groups “laser associated to andiroba” (3.50), “laser” (3.21) and “andiroba” (3.07). On day 8, the groups “positive control” (3.28) and “laser associated to andiroba” (2.57) presented higher means in comparison to groups “laser” and “andiroba” (2.35 and 2.42, respectively). On days 12 and 15, there was no significative statistical difference between groups, however the groups “andiroba” and “laser” remained with the lower score means in comparison the other groups (Table 3) (Figure 3).

**Table 3:** Description of the histopathological analysis to oral mucositis in hamsters treated on days 4, 8, 12 and 15

	**Groups**	***N***	**Mean**	**Median**	**SD**	**Variance**	**Minimum**	**Maximum**
D4	ADB^*^	14	3.07	3.00	0.829	0.687	2	4
	LASER^*^	14	3.21	3.00	0.975	0.951	1	5
	ADB + LASER	14	3.50	4.00	0.650	0.423	2	4
	CONTROL	14	4.07	4.00	0.616	0.379	3	5
D8	ADB^*^	14	2.36	2.00	0.633	0.401	2	4
	LASER^*^	14	2.43	2.00	1.016	1.033	1	5
	ADB + LASER	14	2.57	2.00	0.938	0.879	1	4
	CONTROL	14	3.29	3.00	0.726	0.527	2	4
D12	ADB	14	2.64	2.50	1.216	1.478	1	4
	LASER	14	2.29	2.00	0.611	0.374	1	3
	ADB + LASER	14	2.93	3.00	0.917	0.841	1	4
	CONTROL	14	2.14	2.00	0.770	0.593	1	4
D15	ADB	14	2.00	2.00	0.784	0.615	1	3
	LASER	14	1.86	1.50	0.949	0.901	1	3
	ADB + LASER	14	1.71	2.00	0.726	0.527	1	3
	CONTROL	14	1.86	2.00	0.663	0.440	1	3

ANOVA one-way; ^*^ *p* < 0.05; Control (Positive control group); Andiroba (Andiroba group); Laser (laser group); Adb + laser (Andiroba associated laser group); D4 (4 experimental day); D8 (8 experimental day); D12 (12 experimental day); D15 (15 experimental day).

**Figure 4:**
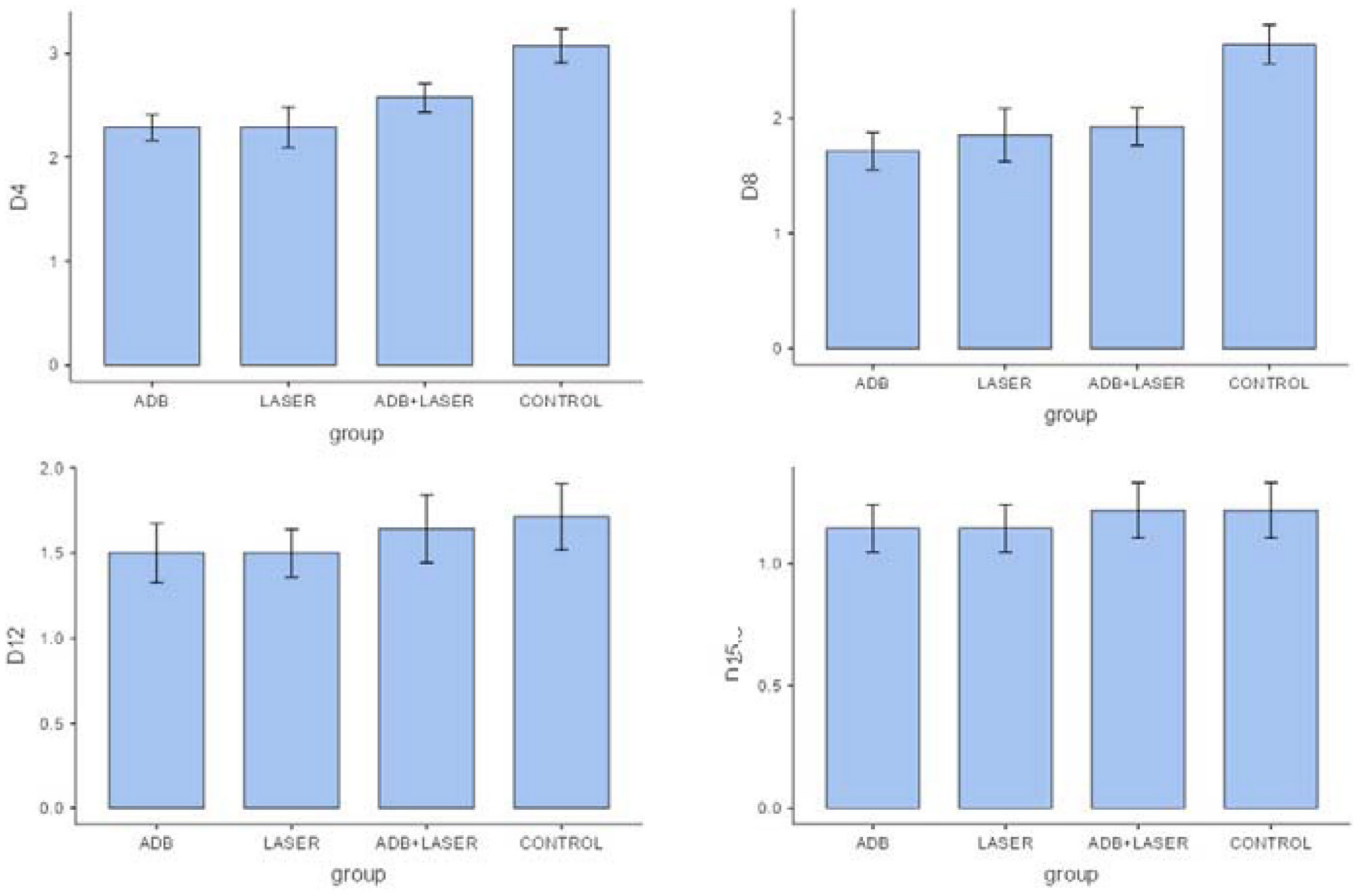
Comparison between clinical analyzes of different groups and days.

**Figure 5:**
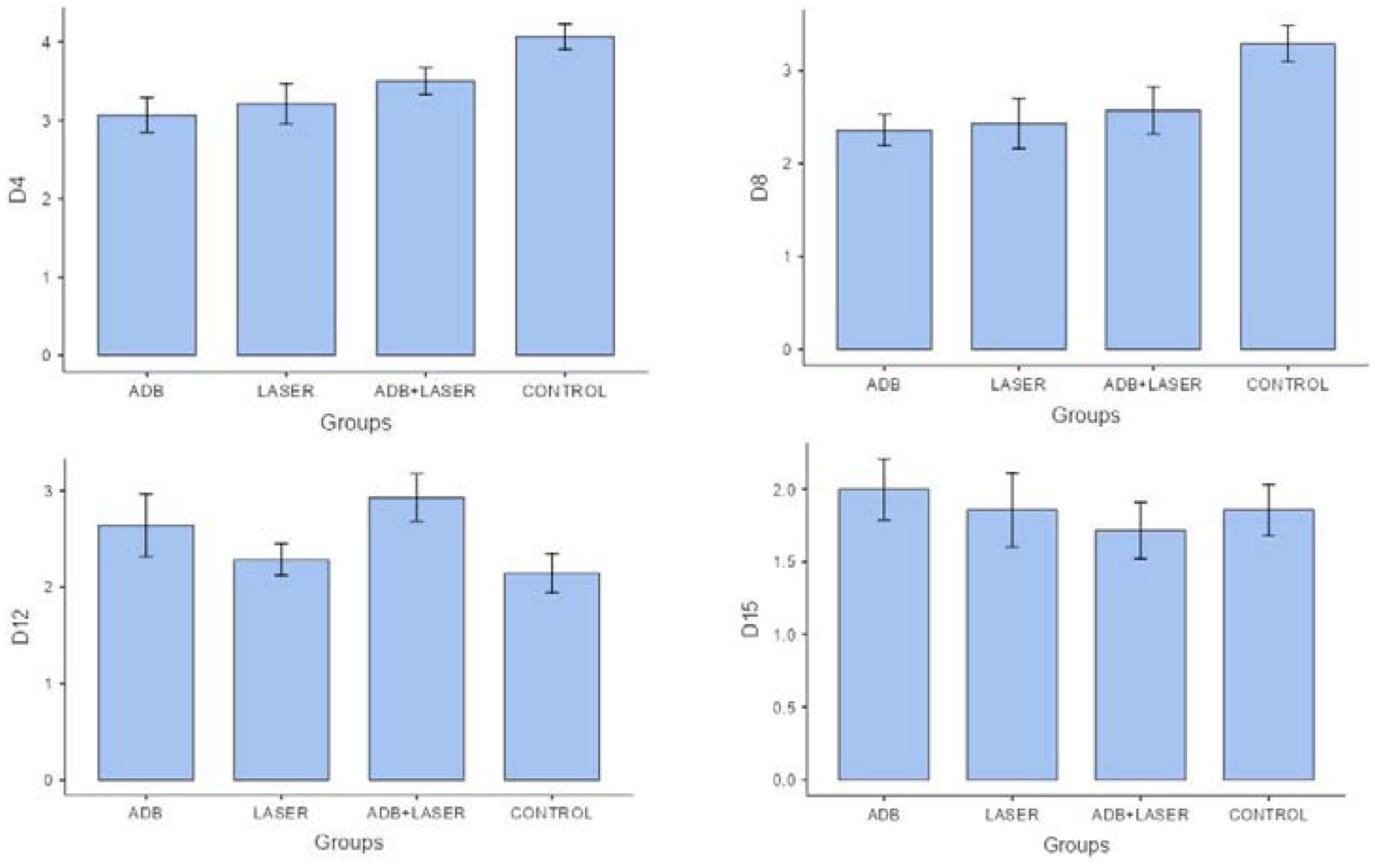
Comparison between histopathological analysis of different groups and days.

The OM decrease on 4,8,12 and 15 days can be observed in Figures 4 and 5.

### Comet test assay

**Figure 6:**
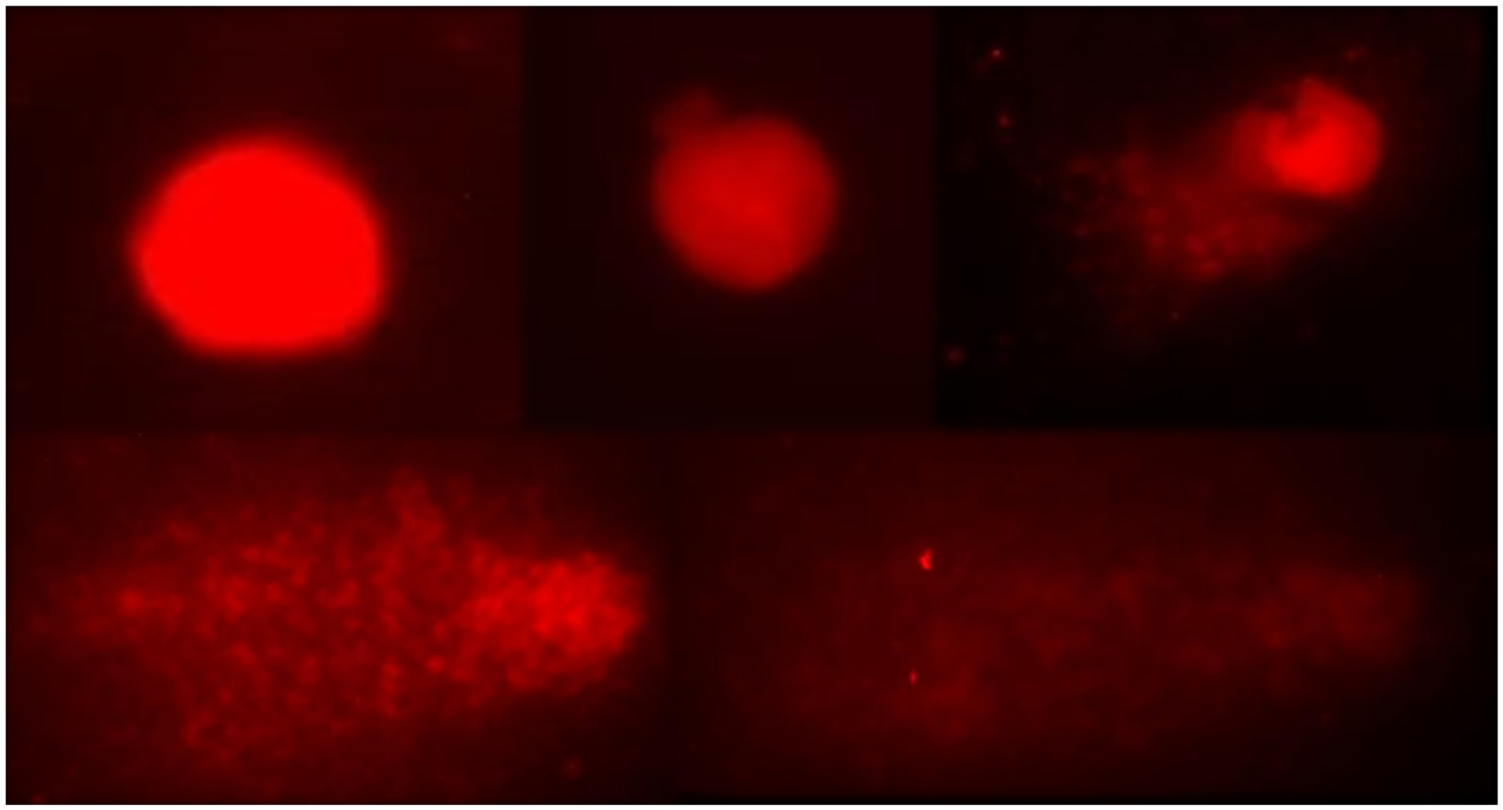
Representation of classes 0 to 4 in the visual classification of comets.

**Figure 7:**
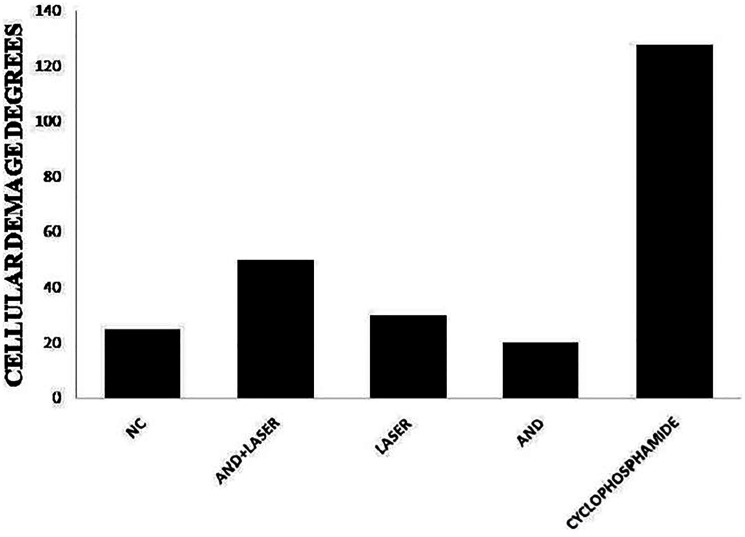
Analysis of the genotoxic effect on the DNA molecules of the cells analyzed in hamsters of the groups submitted to treatments and the cyclophosphamide group (control group).

To evaluate genotoxicity, an *in vivo* comet test was performed, which was followed by a third previously trained examiner analysis of the glass slides. The method applied for this study was based on the guidelines of Organization for Economic Co-operation and Development (OECD). After the counting of cells, a damage index was established (DI) and a final mean is obtained for each sample. The calculation of DI was made by the sum of the products from the score with the number of damages regarding to each level (Figure 6). For better comprehension of the test results, we provide the Table 4. To clearly observe these results, the Figure 7 is combined with a graphic of the DI means of the groups in matter.

**Table 4:** Observation the DI (damage index) to the DNA molecule in different groups

**Groups**	**Average**	**Standard Deviation**
**NC**	24.62	1.88
**LASER**	30.37	7.72
**ADB**	19.16	2.08
**ADB + LASER**	47.83	8.09
**CICLOPHOS**	125.83	10.53

Abbreviations: NC: Negative Control Group; CICLOPHOS: Cyclophosphamide Group; LASER: Laser Group; ADB: Andiroba Group; ADB + LASER: Andiroba group associated with laser.

In Table 4, it is possible to observe the DI to the DNA molecule in different groups. It is also possible to observe an expressive result on cyclophosphamide groups, however that is an expected result, due the fact that this group is a control for the test. The group cyclophosphamide, when compared to group negative control, presented significant statistical difference. The groups andiroba, laser and laser associated to andiroba did not present any significant statistical difference when compared to each other. Although the group andiroba presented lower means (19.16) when compared to the group negative control (24.62), they did not present any significant difference. However, it was observed that the association between laser andiroba treatments, when compared to the control, presented statistical difference (*p* < 0.003), and the association presented expressive means (47.83), when compared to the groups negative control means (24.62).

## DISCUSSION

The evaluation of chemo inducted scarring activity of OM in hamster and the search for efficient treatment with decrease of genotoxicity were the guides for this research. This study was guided by clinical and histopathological investigations about the scarring activity on mucosae of hamsters subjected to grooving and OM induction using chemotherapy drugs (5-Fluorouracila), with posterior treatment by laser and andiroba oil. Besides, to evaluate the use viability of these treatments, their genotoxicity was analyzed, in addition to oil characteristics and its lipidic components for clearer knowledge of its particularities.

The low frequency laser therapy is considered a gold standard in OM, due to its efficiency in the process of healing acceleration, promoting inflammation and pain decrease. The laser luminous energy is converted into useful energy for the cell and is absorbed by chromophores inside mitochondria, essential in the cellular respiratory chain. This process results in the increase of adenosine triphosphate production (ATP), the source of cellular energy, aiding in the proliferation and production of proteins, intensifying cellular mitosis, therefore promoting tissue repair [62] and metabolism acceleration, promoting an anti-inflammatory effect and stimulating the production of collagen and angiogenesis [63–66]. However, due to its high cost, the OM treatment is restricted to a limited number of patients [67–69]. Thus, there is a constant search in the scientific community to enable and ease an accessible treatment for all.

Studies show that this oil presents saponifiable compounds, such as the fatty acids, emphasizing palmitic, oleic, stearic and linoleic acids and unsaponifiable compounds, like limonoids, which present higher visibility on therapeutic effects [36, 37]. This study analyzed the lipidic composition of andiroba oil and it was possible to observe that, in the saponifiable portion, the essential acids presenting more expressive percentages were the oleic (47.33%), palmitic (31.46%), linoleic (8.98%) and stearic (7.12%) oils. Among these, the linoleic oil presents an important role in healing acceleration process, since it is a fundamental component in the collagenase regulation, metalloprotein production and induction of granulation [70, 71].

*In vitro* and *in vivo* studies show benefits of fatty acids present in vegetable oils in wound healing, stimulation of cell proliferation and collagen production. In addition, its use is related to the fatty acids’ antimicrobial, anti-inflammatory and antioxidant action [39–48]. Oleic, linolenic and linoleic acids are known in literature to be initiators of anti-inflammatory mediators [32]. Studies have evaluated the topical application of linoleic acid in pressure ulcers in bedridden patients [72] and, experimentally, in mice with application of oleic or linoleic acid for 16 days [73]. In both studies, positive results were observed, with an acceleration of tissue repair within 48 hours of wound induction. It is believed that the positive results in the healing process associated to these fatty acids occurs through increased production of nitric oxide, which results in overexpression of free radicals, helping inflammatory response, in addition to activating macrophages and fibroblasts, stimulating collagen and keratinocyte production, as well as angiogenesis, providing the acceleration of local re-epithelialization process [74, 75]. Another fatty acid of great importance is arachidonic acid, which copes with the inflammatory cells migration, stimulates elastase, angiogenesis and consequent wound healing [76, 77].

Aiming the excellent effects of andiroba oil, this study evaluated its scarring potential in the OM treatment, in which can be observed that group “andiroba” and group “laser” presented statistical difference (*p* < 0.05) when compared to group “control” in histopathological and clinical analysis, on the 4th day of experiment. In this period, several injuries are clinically found with intense hyperemia and erythema, presenting hemorrhage, extensive ulcers and abscesses in some cases. At microscopic level, the injuries presented moderate to intense cellular engorgement, intense cellular infiltration, with polymorphonuclear leucocytes predominance. Thus, hemorrhagic areas, edemas with extensive ulcers predominance, and, in some cases, abscesses. The best result in the OM treatment was obtained by group “andiroba”, with laser treatment showing similar results, even though the associated “laser and andiroba” treatment presented higher means, showing more intense clinical injuries. This study agrees to Wanzeler et al. [27], whom, using clinical and histopathological evaluation of OM under treatment with several andiroba concentrations, observed an intense inflammatory process on 4º day of experiment, with extensive ulcers and presence of abscesses, and the group “andiroba” presented expressive results in comparison to group control.

On day 8, it is possible to notice, by the clinical images of the injuries, intense hyperemia and erythema, hemorrhage and small ulcers (up to 1 cm diameter), and yet the presence of scarring tissue and absence of abscesses. In microscopic analysis, it can be observed moderate presence of vascular engorgement with vacuolation, moderate to intense cellular infiltration with mononuclear leucocytes predominance, presence of hemorrhagic areas, edemas, and, in some cases, ulcers/abscesses. It is possible to observe that the means of andiroba and laser treatments, when isolated, remained low in comparison to the association of andiroba and the control groups. The “laser” and “andiroba” treatments presented the best results. In this scenario, studies regarding OM treatment, using similar therapeutic methods, also presented positive results and partial decrease in the use of topical andiroba [27, 30] and laser [78], both on 8th day of treatment, in agreement to this study, with decrease injury intensity, suggesting the acceleration of the healing process.

The data of this study regarding the laser therapy agree to data presented by literature, which can be explained by the increase in cellular division promoted by the laser, provoking the tissue regeneration, stimulation of fibroblast production and then, softening the OM peak gravity, reducing ulcers duration and promoting pain relief, which are essential in the healing process [78]. Thus, it is possible to comprehend the reason why on days 12 and 15 of experiment, the laser group presents expressive scarring results, with advanced process of tissue repair. The andiroba presented slightly better results than the laser, demonstrating its powerful anti-inflammatory and scarring actions.

The scientific literature demonstrates a relation between wound healing in injured tissues and the intracellular inhibition of nitric oxide (NO) [79]. These factors induce anti-inflammatory agents production and, consequently, inflammation modulation [80]. The limonoids contained in andiroba inhibit the production of nitric oxide induced by liposaccharides and inhibitors of macrophage activation [81, 82]. The effects of low power laser irradiation on cells are mediated by nitric oxide [83]. These factors can be corroborating to the similar healing results in andiroba group and laser group, isolated. The association of laser and andiroba did not show a promising effect, maybe because it could be inducing a dysregulated expression of nitric oxide. One study evaluated the acceleration of tissue repair in mice and related this efficacy to the increase in nitric oxide production, inflammatory pattern improvement, collagen production and subsequent re-epithelialization [84].

The toxicological and pharmacological research conduce the viability to use or not some phytotherapy products [85]. The evaluation of genotoxicity (comet assay) in this study demonstrated that andiroba oil treatment, without association, presents statistically significant difference when compared to the control group (cyclophosphamide) (*p* < 0.0001) and absence of DNA damage. The laser therapy presented no significant damage. These results corroborate with an experimental study, that evaluated andiroba oil use in Wistar rats, inducing acute and subacute toxicity, and reported no toxicity (no DNA damage), showing the therapeutic viability of andiroba oil’s use. [36, 86]. In this study it was also possible to observe that the association of treatments with laser and andiroba presented expressive genotoxicity when compared to control group, demonstrating a different result from treatments laser and andiroba, separately. However, no scientific evidence in agreement to such results has been found.

Thus, it is possible to conclude that both andiroba and laser are efficient treatments in the scarring potentiating effect in experimental models of chemo inducted OM, and andiroba group shows positive and similar results to the laser group for OM treatment, for being a low-cost and easy applied method. In contrast, the association of andiroba and laser, even though presenting better results than control group, presented inferior results, when compared to the groups using andiroba and laser separately. In conclusion, the use of andiroba oil and laser did not show genotoxic potential, when applied separately. However, genotoxic effects due to their association cannot be discarded.

## MATERIALS AND METHODS

### Animals

We used 122 male golden Syrian hamsters (*Mesocricetus auratus*) for this study, with 90 days of age, weighting between 90 and 120 grams, from the Evandro Chagas Institute vivarium, Belém, Pará (PA), Brazil. The hamsters were kept in cages on the vivarium at the CESUPA, under controlled temperature (20°–24°C), relative air humidity (40–70%) and light (“12 h light /12 h dark” cycle). The animals had free access to food and water. The hamsters selection for the experiments was based on the easiness of observing and exposing their jugal mucosa, also, on their tolerance to chemotherapy drug doses for the induction of OM, without a high mortality rate.

### Acquisition of andiroba and gaseous chromatography/mass spectometry (GC-MS) analysis

The andiroba’s oil used in this study belongs to the National Forest of Tapajós, located in west of Pará State (PA), comprehending the cities of Belterra, Aveiro, Rurópolis and Placas. It shares land borders with Tapajós river, with Santarém-Cuiabá road (BR-163) and with Cupari river, and coordinates 3° 31’ 1” S, 55° 4’ 23” W. Inside the forest, this oil is produced at the Comunidade Nossa Senhora do Rosário, Vila Santa Fé in km 200 north and km 67 of the BR-163 on Uruará-PA municipality. The community integrates the Sementes da Floresta Agroextrativist Association and their register is granted by Brazilian Company of Agroextrativist Researches of Brazil (BCAR). The BCAR company provided a technical report securing the quality of andiroba used.

The oil was analyzed by GC-MS using a gas chromatograph (Varian CP 3380 model) equipped with ions detector and capillary column CP-Sil 88 (60 m length, 0.25 mm internal diameter, 0.25 μm film thickness; Varian Inc., USA). This protocol promotes the conversion of fatty acids inside the oil into methyl ester fatty acids (MEFAs). The column temperature was adjusted to 80°C during 4’ and risen to 205°C in a 4°c/min rate. It was applied the Varian Star 3.4.1 software to quantify the fatty acids, with confection of chromatograms and mix of standard fatty acids (Nu-check-prep, Inc., USA). Fatty acid values were quantified in relative percentages of total acids.

### Experimental groups

This study used a total of 122 animals, which were randomized and divided into 6 groups: Group “andiroba oil 100%”, Group “laser associated to andiroba oil 100%”, Group “laser”, Group “positive control”, Group “negative control” and Group “cyclophosphamide” (control group for genotoxicity analysis). The groups andiroba 100%, laser, laser associated to andiroba 100% and the positive control group presented a *n* = 28, each. The groups “negative control” and “cyclophosphamide control” had *n* = 5, each.

In the groups “andiroba oil”, “laser”, “laser associated to andiroba” and “positive control”, the OM was induced by the administration of chemotherapy drug 5-Fluorouracila (Fluoro-Uracil^®^ 250 mg/10 ml, ICN Farmacêutica Ltda.) and a mechanical trauma on jugal mucosa of the animals, followed by their respective treatments from third to the fifteenth day. The “andiroba oil” group and “laser associated to andiroba oil” received the treatment three times a day (each oral mucosa) and approximately 0,5 ml per application (1,5 ml a day). The measurement and standardization required a plastic Pasteur pipette (0,5 ml) and the oil application was made using a plastic-wrapped cotton swab. Water and food were suspended for 1h for higher medication absorption. The laser group had applications of lasertherapy, once a day, without any kind of food restriction. In the group “laser associated to andiroba”, it was performed the application of laser once a day, and afterwards, it was applied andiroba oil three times a day, with food restriction. To perform the laser treatment, the animals were anaesthetized with ketamine in 80 mg/kg dose associated to xylazine in 20 mg/kg dose, by intraperitoneal via. For less risk of bias and in order to reduce the risk of interference from external factors, all groups were exposed to the same daily anesthetic factor.

In group “positive control” (PC), the animals were exposed to OM induction, however, without any kind of treatment. The group “negative control” (NC) did not received any OM induction protocol (absence of mechanical and chemotherapy induction), so their jugal mucosae served as normality standard and were used as negative control for the comet test. The group cyclophosphamide was the control for genotoxicity test (Comet Test) and the chemotherapy drug cyclophosphamide was used in these animals in a 1000 mg/kg dose by gavage 24 h before euthanasia, with the use of syringe and gavage tube.

### Experimental protocol

The OM induction protocol performed on the animals was based on Soris et al. (1990) [87], which aims the most approximate reproducibility of the human body conditions. The administration of chemotherapy drug 5-FU (Fluoro-Uracil^®^ 250 mg/10 ml, ICN Farmacêutica Ltda.) occurred by intraperitoneal injections on the animals, in the days 0, 5 and 10 of experiment, and 60 mg/kg doses of weight.

To a better representation of mucositis, it was performed a mechanical trauma on the oral mucosa of the animals, with previous anesthesia. The mucosa was exposed and attached in the surgical table and, with the aid of a sterile needle of caliber 18, it was performed two linear grooves, on right and left jugal mucosa of each animal, on days 1 and 2 of the experiment. This procedure was performed by only one previously trained operator. On days 4, 8, 12 and 15 seven random animals were selected from each group for photograph sessions and excisional biopsy on right and left jugal mucosae. The samples were submerged in 10% buffered formaldehyde. The euthanasia of the animals occurred by anesthetic overdosage. To perform the Comet Test, bone marrow, liver fragments and blood of the animals were removed on the 15th day, in all groups, including the negative control group.

### Laser phototherapy protocol

The animals were previously anaesthetized and had their mucosae daily exposed for the performance of lasertherapy. The laser applied in this study was the continuous wave diode type (InGaA1P; MM Optics, São Carlos, São Paulo (SP), Brazil) with a wavelength of 660 nm (visible red), spot size of 0.04 cm², irradiance of 1 W/cm², output power of 40 mW, energy density of 6 J/cm², 6 seconds of exposition per spot, in a total of 0.24J, as applied by Weissheimer et al. (2017) [88].

### Jugal mucosae clinical analysis

(The are several typing mistakes at the end of this section. The authors do not mention which clinical and histopathological parameters are going to be evaluated, nor how. Once again, there are problems concerning wording and text clarity).

The clinical evaluation was based on the analysis of severity of OM on the jugal mucosae of the animals, through the photographs taken on days 4, 8, 12 and 15. The images were evaluated by a previously trained examiner and blinded about the groups and treatment.

By performing the images analysis, there was not a complete score framing on the classified images using Lima et al. (2005) scale [61]. However, due to the necessity of more specific scores to the clinical reality of the experiment, it was opted for modifying the table, therefore denominated “Lima modified” – Score 0: absent or discreet hyperemia and erythema, absent hemorrhage, ulcer and abscess absence; Score 1: moderate hyperemia and erythema, absent hemorrhage, ulcer and abscess absence, presence of scarring tissue; Score 2: severe hyperemia and erythema, presence of hemorrhage and small ulcers (up to 1cm diameter), presence of larger area of scarring tissue and absence of abscesses; Score 3: severe hyperemia and erythema, presence of hemorrhage, extensive ulcers and absence of abscesses; and Score 4: severe hyperemia and erythema, presence of hemorrhage, extensive ulcers and abscesses.

### Jugal mucosae histopathological analysis

The analysis was performed by the evaluation of histopathological glass slides which passed through laboratorial processing and were stained for hematoxylin-eosin. The glass slides were codified for the sample blinding, and the evaluation was performed by a third previously trained examiner.

During the histopathological analysis of inflammatory alterations, there were difficulties in the alteration framings in the criteria proposed by Lima et al. (2005) [61]. Due to it, we proposed a new table for the classification on inflammatory and scarring processes, which we denominated “Lima modified” – Score 0: epithelial and connective tissues with no vasodilation, absent or discreet inflammatory cellular infiltrate, absence of hemorrhage, edemas, ulcers or abscesses; Score 1: discreet vascular engorgement, reepithelization areas, discreet cellular infiltration, presence of mononuclear leukocytes, absence of hemorrhage, edemas, ulcers or abscesses; Score 2: moderate vascular engorgement, hydropic epithelial degeneration (vacuolation), discreet cellular infiltration, with mononuclear leucocytes predominance, presence of hemorrhagic areas, edemas, no ulcers and absence of abscesses; Score 3: moderate vascular engorgement, vacuolation, moderate or intense cellular infiltration, with mononuclear leucocytes predominance, presence of hemorrhagic areas, edemas, no ulcers and absence of abscesses; Score 4: moderate vascular engorgement, vacuolation, moderate or intense cellular infiltration, with polymorphonuclear leucocytes predominance, presence of hemorrhagic areas, edemas and eventual small ulcers and absence of abscesses; Score 5: intense vascular engorgement, intense vasodilation, intense cellular infiltration, with polymorphonuclear leucocytes predominance, presence of hemorrhagic areas, edemas, abscesses and extensive ulcers.

### *In vivo* comet test

To evaluate the genotoxicity of treatments with laser and andiroba oil, as well as their association, during the 15 experimental days, a comet test was performed. The test was performed on the 15th day of experiment, in a sample of 30 animals, being 5 from each experimental group – (a) laser; (b) andiroba; (c) laser associated to andiroba; (d) negative control; (e) Cyclophosphamide, in which the animals were submitted to application of cyclophosphamide in a 1000 mg/kg dose by gavage 24 h before euthanasia, with the aid of syringe and gavage tube. For the test’s performance, the animals were “euthanized” and had their femur (bone marrow) removed, using a syringe (5 ml) and fetal bovine serum. Besides, blood was collected directly from the animal hearts and liver fragments.

The glass slides were previously covered in agarose solution in 1.5% normal melting point. The liver was macerated and mixed to the blood and bone marrow, until thorough mixing. After complete sample procedure, the glass slides remained in the lysis solution, in low luminosity environment, in which they were arranged in horizontal position in the electrophoresis tub, set in a 34V voltage in 300 mA electrical current for 20 minutes. The glass slides were, then, removed from the tub and submerged in icy distilled H_2_O (4°C) for electrophoresis solution removal. The washing procedure was repeated; however, the glass slides were immersed for 5 minutes for neutralization. The glass slides were fixed by being immersed in absolute ethanol for 3 minutes. After that, they were stained with 50 μL Ethidium bromide (20 μL) and covered in glass slide for analysis.

The glass slides were analyzed in duplicate of each sample, using a fluorescence microscope Olympus BX41 model (Olympus Co., Japan), and in each glass slide 50 cells were counted. The analysis was performed by the score standard, in which, according to size and intensity of the comet’s tail, the scores are classified in 5 categories, with variant damage of 0–4, according to the percentage of DNA in the comet’s tail, which indicates the injury degree suffered by the cell, as proposed by Singh et al. (1988) [89].

### Statistical analysis

The sample size was determined by pilot study using the ANOVA test, in which it was adopted 0.05 of significance level and 80% power of evidence. Statistical tests of variance analysis (ANOVA) or Kruskal Wallis were performed, depending of sample normality distribution. In significant difference cases between groups, Tukey post-test (histopathological analysis and comet test) and Dunn post-test (Clinical Analysis) were applied. The significance level was established in 5% (*p* < 0.05) in all tests. Bioestat software, version 5.0 was used to perform such tests.
