# Addendum: The chromatographic constitution of andiroba oil and its healing effects, compared to the LLLT outcomes, in oral mucositis induced in golden Syrian hamsters: a new treatment option

**DOI:** 10.18632/oncotarget.28504

**Published:** 2023-09-22

**Authors:** Jessica T. Gomes, Ana Márcia V. Wanzeler, Sergio M.A. Júnior, Rosa Helena F. Chaves Soares, Carolina P. de Oliveira, Emanuelle de M. Rodrigues, Bruno M. Soares, Diego D.F.A. Alcantara, Rommel M.R. Burbano, Fabrício M. Tuji

**Affiliations:** ^1^Department of Dentistry, Federal University of Pará, Guamá, Belém, Pará 66075-970, Brazil; ^2^Department of Medicine, University Center of Pará, Souza, Belém, Pará 66613-903, Brazil; ^3^Department of Genetics, Laboratory of Human Cytogenetics, Federal University of Pará, Guamá, Belém, Pará 66075-970, Brazil

**This article has an addendum:** Acknowledgements - To the Experimental Research Group and the Laboratory of Histopathology of the State University of Pará (CESUPA) for the technical assistance provided and to the Laboratory of Human Cytogenetics of the Federal University of Pará (LCH/UFPA). This study was also supported in part by scholarships for postgraduate students of Coordination for the Improvement of Higher Education Personnel - Brazil (CAPES) - Finance Code 001. To the support of PROPESP/UFPA (PAPQ) (23073.074981/2022-97).

Original article: Oncotarget. 2023; 14:23–39. 23-39. https://doi.org/10.18632/oncotarget.28338

